# Supplementary material for: Deuterated buprenorphine retains pharmacodynamic properties of buprenorphine and resists metabolism to the active metabolite norbuprenorphine in rats
Source: Front Pharmacol. 2023 May 9;14:1123261. doi: 10.3389/fphar.2023.1123261 (PMC10204800; doi:10.3389/fphar.2023.1123261)
Supplement: Supplementary file 1 [file Table1.DOCX]

**Supporting Data**

Figure 1.

A.

1H-NMR spectroscopy of BUP-D2

B.

13C-NMR spectroscopy of BUP-D2

Figure 2.

MRM chromatogram of 100 ng/mL D2 buprenorphine (top is transition for Bup m/z 468.4🡪396.1 and bottom is transition for BupD2 m/z 470.4🡪398.3).
